# Primary Uterine Nongestational Placental Site Trophoblastic Tumor as a Distinct Entity: A Report of 5 Cases

**DOI:** 10.1097/PAS.0000000000002502

**Published:** 2026-01-06

**Authors:** Alexis Trecourt, Geoffrey J. Maher, Rosemary A. Fisher, Katie McDonald, Michael J. Seckl, Matthew C. Winter, Snezana Susnjar, Vesna Kesić, Pierre Descargues, Mojgan Devouassoux-Shisheboran, Fabienne Allias, Baljeet Kaur

**Affiliations:** * Hospices Civils de Lyon, Centre Hospitalier Lyon-Sud, Service de Pathologie Multi-Site, Pierre Bénite; † Université Claude Bernard Lyon-1, Faculté de Médecine Lyon Sud, Centre Pour l’innovation en Cancérologie de Lyon (CICLY), Lyon, France; ‡ Trophoblastic Tumour Screening & Treatment Centre, Imperial College NHS Trust, Charing Cross Hospital; § Department of Surgery and Cancer, Imperial College London, Charing Cross Hospital, London; ∥ Sheffield Centre for Trophoblastic Disease, Weston Park Cancer Centre, Sheffield Teaching Hospitals NHS Foundation Trust, Sheffield; # Division of Clinical Medicine, School of Medicine and Population Health, University of Sheffield, Sheffield, UK; ** Department for Medical Oncology, Institute for Oncology and Radiology Belgrade; ‡‡ Hospices Civils de Lyon, Centre Hospitalier Lyon-Sud, Service de Chirurgie Gynécologique, Pierre Bénite; §§ Centre Français de Référence des Maladies Trophoblastiques; ∥∥ Université Claude Bernard Lyon-1, Faculté de Médecine Lyon-Est, Lyon, France; ¶¶ Department of Pathology, North West London Pathology, Imperial College NHS Trust; ## Department of Metabolism, Digestion and Reproduction, Imperial College London, Charing Cross Hospital, London; ¶ Histopathology Department, South Yorkshire and Bassetlaw Pathology, Royal Hallamshire Hospital, Sheffield, UK; †† Department of Human Reproduction, Medical Faculty, University of Belgrade, Belgrade, Serbia

**Keywords:** PSTT, nongestational trophoblastic tumor, uterine neoplasm, trophoblastic differentiation, STR genotyping

## Abstract

Uterine placental site trophoblastic tumors (PSTTs) are rare trophoblastic neoplasms, presumed to be of gestational origin. Herein, using a comprehensive morphologic, immunohistochemical, and molecular approach, we describe 5 cases of primary uterine nongestational PSTTs. The median age at presentation was 32 years (range 25 to 45). All tumors were initially expected to be of gestational origin as all were located in the uterus and all patients had a history of pregnancy (5/5, 100%). The median size of the primary uterine tumors was 6.3 cm (range 4.8 to 7.5). Three patients (3/5, 60%) had metastatic disease at presentation or revealed during initial workup (1/5 [20%] patients with lymph node metastasis only and 2/5 [40%] with distant metastases). All tumors showed similar histopathologic and immunohistochemical features to those of gestational PSTTs. The tumor cells expressed hPL in 5/5 (100%) tumors, hCG in 5/5 (100%; focal in all tumors), and GATA3 in 5/5 (100%). However, short tandem repeat (STR) genotyping did not identify any nonpatient alleles in the tumors, indicating a nongestational origin. The median progression-free survival was 18 months (range: 0 to 85) and 2/5 (40%) patients died from disease, highlighting the potential poor prognosis of this nongestational tumor. Thus, in the same way as gestational and nongestational choriocarcinomas are recognized as different entities, nongestational PSTTs could be viewed as a distinct entity from their gestational counterparts, although further investigation and more cases are needed. Furthermore, we propose recommendations for diagnosing and staging of nongestational PSTTs to improve patient stratification and management.

In the *World Health Organization (WHO) Classification of Female Genital Tumours*, 5th edition,^1^ gestational trophoblastic neoplasia (GTN) comprises gestational choriocarcinoma, placental site trophoblastic tumor (PSTT), epithelioid trophoblastic tumor (ETT), and mixed trophoblastic tumor. Whereas the putative cell of origin for gestational choriocarcinoma is chorionic villous trophoblast, PSTTs and ETTs are thought to derive from implantation site intermediate trophoblast (ISIT) and chorionic-type intermediate trophoblast (CTIT), respectively.^1^ Even though PSTTs and ETTs are the rarest subtypes of gestational trophoblastic disease (GTD), they account for the majority of deaths.^2^

Due to their gestational origin, the genome of GTN reflects that of the causative pregnancy and not that of the patient, a feature which distinguishes GTN from all other somatic and germ cell malignancies.^3,4^ Short tandem repeat (STR) genotyping is therefore recommended to distinguish between gestational choriocarcinoma, nongestational choriocarcinoma, and other somatic malignancies with trophoblastic differentiation, as they require different clinical management.^5^ Although STR genotyping is not always performed worldwide for uterine ETTs and PSTTs, which are presumed to be of gestational origin, it enables differential diagnosis of these tumors from somatic tumors, especially in cases of extra-uterine involvement where GTN mimics have been reported.^6,7^ STR genotyping can also provide information on the interval between tumor diagnosis and the antecedent/causative pregnancy, which is a key prognostic factor for PSTTs and ETTs, where an interval of ≥48 months is associated with poor prognosis.^1,2,8^ While PSTTs and ETTs with a pregnancy interval of <48 months that are restricted to the uterus are generally cured by surgical resection, adjuvant chemotherapy and/or immunotherapy is recommended for an interval of ≥48 months and tumors that have metastasized.^8–10^

Here, we report for the first time the identification of 5 intramyometrial nongestational PSTTs occurring as primary uterine tumors. In addition to characterizing this histopathologic and genetic tumor, we highlight the important implications for clinical practice.

## MATERIALS AND METHODS

### Case Selection, Clinical and Follow-Up Data Collection

As part of routine pathologic and genetic diagnosis at GTD reference centers, genotyping of primary uterine PSTTs identified tumors whose genotype reflected that of the patient, indicating a nongestational origin. Two cases (#1 and #4) were discussed during a pathologists’ and geneticists’ multidisciplinary team meeting of the European organization for treatment of trophoblastic diseases (EOTTD).^11^ Three previously identified cases of primary uterine PSTT with nongestational genotypes reviewed at the same GTD reference centers were incorporated for a total of 5 cases. For each patient, the following information was retrieved from the pathologic reports and local electronic medical databases: the age at presentation, the serum human chorionic gonadotrophin (hCG) level at presentation, the obstetrical and past medical/surgical history, the clinical symptoms at presentation, the tumor location (primary site and extrauterine involvement[s]), the tumor extension, the tumor size, the type (curettage material, surgical specimens) and location of the sample which allowed the first histopathologic diagnosis, the time interval from end of pregnancy to PSTT diagnosis, the international federation of gynecology and obstetrics (FIGO) staging (using both the FIGO staging for GTN^12^ at diagnosis and, retrospectively, the FIGO staging for nongestational tumor of the uterus^1^ after the STR genotyping results), the surgical and medical treatment, the follow-up duration (until May 2025), recurrence(s), and the most recent status. The median follow-up and median progression-free survival (PFS) were calculated. This study complies with the Declaration of Helsinki.

### Histopathologic Analysis

All hematoxylin-eosin-saffron (HES)-stained slides, hematoxylin-eosin (H&E)-stained slides, and immunohistochemistry slides, prepared before the present study for diagnostic purpose, were reviewed by trophoblastic disease and gynecological expert pathologists to ensure the diagnosis of PSTT, according to the *WHO Classification of Female Genital Tumours*, 5th edition.^1^ For all tumors, the following histopathologic criteria were noted: the tumor delimitation with adjacent myometrium, the tumor architecture, vascular invasion with replacement of the vascular wall and fibrinoid necrosis, tumor necrosis, hemorrhagic changes, multinucleated cells, pleomorphic cytologic atypia, lymphocytic infiltrate (mild, moderate, severe), atypical mitoses, and other trophoblastic or nontrophoblastic neoplasia. The mitotic count was also specified per 10 high-power fields (HPFs).

### Immunohistochemical Analysis

Immunohistochemical staining was performed for most cases using the following antibodies: anti-hCG, anti-hPL, anti-GATA3, anti-Ki67, anti-p53, and anti-p63, and a subset of tumors with the following antibodies: anti-AE1/AE3, anti-CAM 5.2, anti-CK18, anti-p40, anti-inhibin, anti-desmin, anti-caldesmon, anti-smooth-muscle actin (SMA), anti-melanA, anti-HMB45, anti-SALL4, anti-p53, anti-PAX8, anti-estrogen receptor (ER), anti-progesterone receptor (PR), anti-PLAP, anti-MCAM (CD146/Mel-CAM), anti-CD10, anti-MLH1, anti-MSH2, anti-MSH6, anti-PMS2, anti-PD-L1. Results were reported as negative (−; with a specification as “occasional cells” in case of occasional positive cells) or positive (+; defined as extent of staining ≥10%, with a specification as “focal” in case of staining near to 10% of tumor cells, both focal and ≥10% hPL staining being consistent with the diagnosis of PSTT). For p53, the wild-type/mutant-type status was reported, and for mismatch repair (MMR) proteins (MLH1, MSH2, MSH6, PMS2), the proficient MMR status (pMMR) or deficient MMR status (dMMR) were reported. For Ki67 and PD-L1, the proportion (%) of tumor cell positivity was indicated.

### Molecular Genotyping

STR genotyping was performed for all cases, with genotypes of the tumor compared with those of the patient (adjacent normal tissue, blood or buccal cells). In 3 cases, genotypes were also compared with those of the patient’s partner and child (cases #2 and #4) or the patient’s partner (case #5). Using HES/H&E-stained sections as a guide, one or more tumor regions with ≥30% cellularity were manually dissected from adjacent unstained formalin-fixed paraffin-embedded (FFPE) sections. DNA from FFPE tissue samples was extracted using a QIAamp DNA FFPE Tissue Kit (Qiagen, Germantown, MD) according to the manufacturer’s instructions. DNA was extracted from blood using a QIAamp Blood Mini Kit (Qiagen) according to the manufacturer’s instructions. DNA was extracted from buccal cells using a GeneJET Genomic DNA Purification Kit (ThermoFisher Scientific, Waltham, MA) or Oragene prepIT L2P (DNA Genotek, Ontario, Canada), according to the manufacturer’s instructions.

For each case, a minimum of 15 STR loci and the Amelogenin (sex) locus were amplified using the AmpFLSTR Identifier Plus kit (Applied Biosystems, Carlsbad, CA) and/or the GlobalFiler PCR Amplification Kit (Applied Biosystems), and/or the VeriFiler Express PCR Amplification Kit (Applied Biosystems), according to the manufacturer’s instructions. PCR products were resolved by capillary electrophoresis, and genotypes were analyzed in GeneMapper version 5.0 (Applied Biosystems). Only allelic imbalances that were present in all analyzed regions of a tumor, but not in the matched normal tissue, were reported.

For tumors #3 and #4, targeted capture and sequencing of a custom panel of 316 common single-nucleotide polymorphisms (SNP), 15 chrX targets, and 8 chrY targets was also performed. Libraries were made using the DNA previously extracted for STR genotyping, using xGEN DNA Library Prep EZ UNI library preparation kits (IDT, Coralville, IA) and Cell3 Target capture probes (Nonacus, Birmingham, UK). Paired-end Illumina Nextseq sequencing (tumor #3) was performed by the NIHR Imperial BRC Genomics Facility, and Novaseq sequencing (tumor #4) was performed by Genewiz UK Ltd (Essex, UK) with a minimum mean consensus read depth of 1313 per tumor and 4411 per patient. Loci that were homozygous in the patient's DNA were investigated in the tumor DNA for the presence of nonpatient/paternal alleles, as previously described.^13^

### DNA Targeted Next-Generation Sequencing (NGS)

Targeted high-throughput sequencing of 100 solid tumor-associated genes was performed for tumor #1 using the Sophia Genetics “Solid Tumor Solution” pan-cancer panel (Sophia Genetics, Lausanne, Switzerland) as previously described^14^ (Supplemental Table S1, Supplemental Digital Content 1, http://links.lww.com/PAS/C215). The library was sequenced on a NextSeq 500 (Illumina) to a minimum depth of 150×. Variants were interpreted using the SophiaDDM v4 interface with OncoPortal (SophiaGenetics). Only likely-pathogenic and pathogenic variants (pathogenicity class 4 and 5, respectively, according to the American College of Medical Genetics) were retained during the analysis.

### Targeted RNA-Sequencing

For tumor #1, RNA was extracted from FFPE tissue using a Maxwell 16 LEV RNA FFPE purification kit (Promega, Madison, WI) on a Maxwell 16 instrument (Promega). Fifty nanograms of RNA was used for library preparation and capture using probes targeting 156 genes (Supplemental Table S2, Supplemental Digital Content 2, http://links.lww.com/PAS/C216) was performed using “Custom RNA target Bundle Solution” (SOPHIA RNA Library Preparation Kit IV, Sophia Genetics) as previously described.^15^ Libraries were sequenced on an Illumina NextSeq 500 sequencer (Illumina) with >3 million reads. Interpretation was performed using the RCROS_A_v1 pipeline (Sophia Genetics) for fusion transcript calling.

### Comparative Genomic Hybridization Analysis (aCGH)

aCGH was performed for tumor #1, using the DNA previously extracted, the CYTAG SuperCGH Labeling Kit (Enzo Life Science, Villeurbanne, France) and the SurePrint G3 Human Genome CGH+SNP Microarray, 4x180K (Agilent Technologies, Santa Clara, CA), according to the manufacturer’s instructions. Data analysis was carried out using the Cytogenomics software (Agilent Technologies; v5.4.0.11), which used the following parameters: ADM-2, threshold: 5.0, window: 1 Mb.

### Statistical Analysis

Descriptive statistics were expressed as counts and proportions for dichotomous variables, and medians and ranges for continuous values.

## RESULTS

### Clinical and Laboratory Data

Following pathologic reviews and genetic analyses at GTD reference centers, the 5 cases were confirmed to be nongestational uterine PSTTs. The median age at presentation was 32 years (range: 25 to 45). All tumors were located in the uterus, and the median size was 6.3 cm (range: 4.8 to 7.5). Whole-body imaging at the time of presentation revealed distant metastases in 2 patients (#3 and 5). Clinical data are provided in Table 1 and Supplemental Table S3, Supplemental Digital Content 3, http://links.lww.com/PAS/C217.

**TABLE 1:** Main Clinical Data and Histopathologic Features of Nongestational Placental Site Trophoblastic Tumors

	Patient/tumor #1	Patient/tumor #2	Patient/tumor #3	Patient/tumor #4	Patient/tumor #5
Age at diagnosis	34	28	25	45	32
Pregnancy history	G1P1	G2P1	G1P0	G1P1	G3P3
hCG level at presentation (mIU/mL)	74	330	319	7	6571
Location	Uterine fundus and right uterine horn	Right cornua of the uterus	Uterus	Uterus	Uterus
Tumor size (cm) at diagnosis	6.3	4.8	7.5	6.9	4.8
No. samples performed per tumor (paraffin blocks)	7	6	>1	16	12
FIGO stage at diagnosis for GTD	I	I	IV	II	I (and then IV)
FIGO for nongestational uterine tumor	IIIC	IIIA	IVB	IIIB	IIIC (and then IVB)
Morphologic features (HES/H&E)	Typical features of PSTT	Typical features of PSTT	Typical features of PSTT (but with high mitotic count and pleomorphic nuclei)	Typical features of PSTT	Typical features of PSTT
Neoadjuvant treatment	None	EP/EMA, PAC-E PAC-PLT MTX	Low dose EP, MTX	None	None
Hysterectomy performed?	Yes	Yes	No	Yes	Yes
Adjuvant treatment	EP/EMA	Pembrolizumab	None	EP/EMA	EP/EMA followed by TP-TE
Metastasis or distant involvement	Right pelvic lymph node	None	Lung, liver, lymph nodes, breast, diaphragm, kidney, pericardium	Left parametrial involvement	Retroperitoneal and pelvic lymph node metastases, and then bone metastases (pubis)
Follow-up time (mo)	36	85	<1	18	18
Relapse, outcomes after treatment completion	None, NED	None, NED	DOD	None, NED	DOD
Progression-free survival (mo)	36	85	0	18	NA

# indicates number; DOD, died of disease; EP/EMA, etoposide and cisplatin, and then etoposide, methotrexate, and dactinomycin; FIGO, International federation of gynecology and obstetrics; GTD, gestational trophoblastic disease; GxPx, gravidity x and parity x; H&E, hematoxylin & eosin; HES, hematoxylin-eosin-saffron; MTX, methotrexate; NA, not available; NED, no evidence of disease; PAC-E, cisplatin, doxorubicin, cyclophosphamide, etoposide; PAC-PLT, Cisplatin, Doxorubicin, Cyclophosphamide, and platinum; PSTT, placental site trophoblastic tumor; TP/TE, paclitaxel/cisplatin alternating with paclitaxel/etoposide.

Patient #1 was a 34-year-old G1P1 woman, who presented with metrorrhagia and amenorrhea for 10 months after her full-term uncomplicated cesarean delivery. Serum hCG level was elevated (74 mIU/mL). The patient had a bicornuate uterus and Beckwith-Wiedemann syndrome due to loss of methylation at the imprinting control region 2. Endometrial curettage was performed and a diagnosis of PSTT was made. Magnetic resonance imaging (MRI) showed a 6.3 cm mass infiltrating more than half of the myometrium at the uterine fundus and at the right uterine horn. She underwent a hysterectomy and sentinel node procedure. The histopathologic diagnosis of PSTT was confirmed on the surgical specimen, and one metastasis in a sentinel node was found. Thus, the patient underwent lumbo-aortic lymph nodes dissection, which showed no other metastasis. She then underwent adjuvant chemotherapy. Post-surgery, the serum hCG level returned to normal 4 weeks later. The patient is alive without evidence of the disease 36 months after the diagnosis.

Patient #2 was a 28-year-old G2P1 woman who presented with metrorrhagia 6 years after her last pregnancy. She reported a positive pregnancy test and her serum hCG level was elevated at 330 mIU/mL. Ultrasound imaging showed a mass located in the right uterine cornua, and the patient underwent a bilateral salpingectomy with partial removal of the cornua. GTN with morphologic features of PSTT was diagnosed, measuring 4.8 cm. Serum hCG remained elevated following surgery and the patient underwent neoadjuvant chemotherapy. Serum hCG normalized and subsequently hysterectomy, and peritoneal and ovarian staging biopsies were performed. After a histopathologic diagnosis of PSTT made on the surgical specimen, which showed a parametrial involvement, she underwent adjuvant immunotherapy (*pembrolizumab*). The patient is alive at 85 months after the diagnosis.

Patient #3 was a 25-year-old G1P0 woman who presented with fatigue, shortness of breath, chest pain, cough, vaginal discharge, and elevated serum hCG (319 mIU/mL), ∼4 years after a termination of pregnancy. An endometrial curettage was performed because of a 7.5 cm uterine mass associated with lung, liver, lymph node, breast, diaphragm, kidney, and pericardium metastases. The patient underwent adjuvant chemotherapy before the diagnosis of PSTT. The patient died from the disease 4 days after the diagnosis. The histopathologic diagnosis of PSTT was then confirmed on extensive postmortem samples from several metastatic locations.

Patient #4 was a 45-year-old G1P1 woman who presented with bloating, fatigue, and irregular vaginal bleeding for 8 months, 22 years after her last normal vaginal delivery. Serum hCG level was 7 mIU/mL. Imaging studies revealed a 6.9 cm uterine mass. She underwent a hysterectomy, bilateral salpingo-oophorectomy, and omentectomy. A histopathologic diagnosis of PSTT was made on the surgical specimen, with left parametrial involvement. The patient underwent adjuvant chemotherapy and is alive without evidence of disease 18 months after the diagnosis.

Patient #5 was a 32-year-old G3P3 woman who presented with a 4.8 cm uterine mass and a serum hCG level of 6571 mIU/mL, 9 years after her last delivery. She underwent a subtotal hysterectomy, left salpingo-oophorectomy, followed by a complete surgery with cervical extirpation. A histopathologic diagnosis of PSTT on the surgical specimen was made, and the patient underwent adjuvant chemotherapy. Whole body imaging revealed necrotic lymphadenopathy with stenosis of the distal third of the right ureter, pathologic lymphadenopathy in the pelvis, and bone secondary deposit within the symphysis of the pubis. The patient died from the disease 18 months after the diagnosis.

### Histopathologic and Immunohistochemical Findings

**FIGURE 1:**
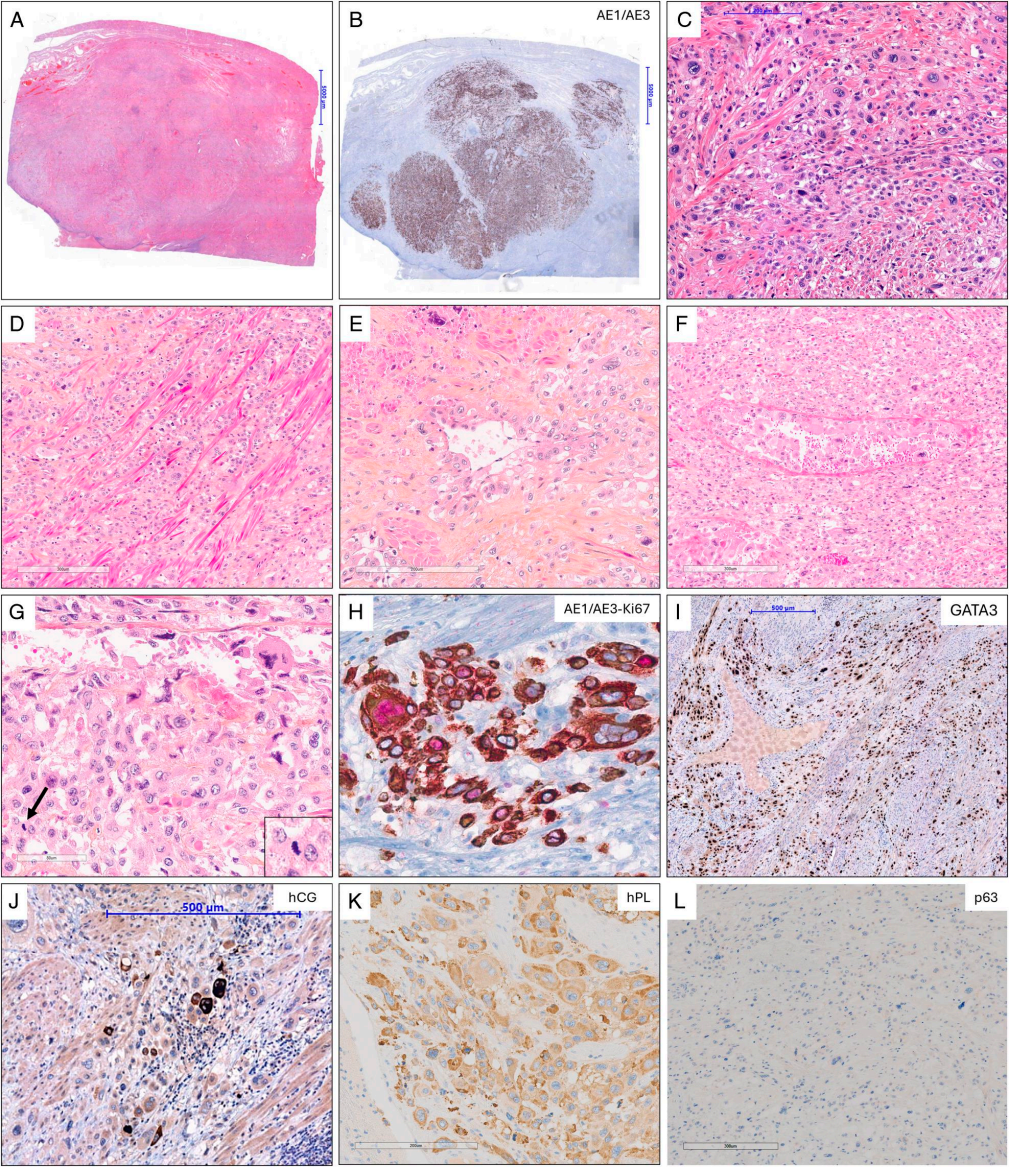
Histopathologic and immunohistochemical features of uterine nongestational PSTTs. A (Hematoxylin-eosin [HE], tumor #4) and B (AE1/AE3 IHC, tumor #4), Uterine tumor with defined borders and >50% myometrial infiltration (low-magnification). C (HE, tumor #4) and D (hematoxylin-eosin-saffron [HES], tumor #1), Sheets and cords of tumor cells infiltrating the myometrium and dissecting smooth muscle bundles without tumor stromal reaction. E (HES, tumor #1) and F (HES, tumor #1), Vascular wall invasion with replacement of the vascular wall and fibrinoid necrosis. G (HES, tumor #1), Tumor cells with abundant eosinophilic cytoplasm and pleomorphic atypical nuclei. Note one mitotic figure (arrow). H (AE1/AE3 IHC [brown]—Ki67 IHC [red], tumor #1), A >10% proliferative index in tumor cells expressing AE1/AE3. I (GATA3 IHC, tumor #4), Diffuse positivity of GATA3 in tumor cells. J (hCG IHC, tumor #4), Focal positivity of hCG in tumor cells, especially multinucleated cells. K (hPL IHC, tumor #1), Diffuse positivity of hPL in tumor cells. L (p63 IHC, tumor #1), Absence of p63 expression by tumor cells. IHC indicates immunohistochemistry; PSTTs, placental site trophoblastic tumor.

All tumors were poorly circumscribed in relation to the adjacent myometrium and showed typical histopathologic features of PSTT (Fig. 1). Sheets and cords of ISIT infiltrating the myometrium and dissecting smooth muscle bundles without tumor stromal reaction were observed in all tumors. In all tumors, tumor cells with abundant eosinophilic cytoplasm and pleomorphic atypical nuclei with nucleoli were observed. Similarly, vascular invasion with replacement of the vascular wall and fibrinoid necrosis, and nonsyncytiotrophoblastic multinucleated cells were observed for all tumors. Tumor necrosis and hemorrhagic changes were observed in 3/4 (75%; focal in one tumor) tumors and 4/5 (80%), respectively. Atypical mitoses were observed in all (5/5, 100%) tumors and the median mitotic count was 6 mitoses per 10 HPFs (range: 2 to 8). No nontrophoblastic neoplasia was observed in the uterus or metastatic sites. The lymphocytic infiltrate was mild in 4/5 (80%) tumors and moderate in 1/5 (20%).

The tumor cells expressed hPL in 5/5 (100%; focal in 2 tumors), hCG in 5/5 (100%; focal in all tumors), and GATA3 in 5/5 (100%). In contrast, p63 was always negative (occasional cells positive in 2 tumors). The p53 status was wild-type in the 2 tumors tested. The median Ki67 proliferative index was 24% (range: 10% to 15% to 40%). The immunohistochemical findings are detailed in Table 2 and Supplemental Table S4, Supplemental Digital Content 4, http://links.lww.com/PAS/C218.

**TABLE 2:** Immunohistochemical Results of Nongestational Placental Site Trophoblastic Tumors

Patient/tumor #	Patient/tumor #1	Patient/tumor #2	Patient/tumor #3	Patient/tumor #4	Patient/tumor #5
hPL	+	+ (focal)	+	+	+ (focal)
p63	−	− (occasional cells)	−	−	− (occasional cells)
hCG	+ (focal)	+ (focal)	+ (focal)	+ (focal)	+ (focal)
GATA3	+	+	+	+	+
SALL4	−	NP	NP	NP	NP
p53	Wild-type	NP	NP	Wild-type	NP
Ki67	18%	10-15%	NP	40%	30%

# indicates number; +, positive; −, negative; NP, not performed.

It is worth noting that for tumor #3, although tumor architecture and immunohistochemical profile were highly suggestive of a PSTT, the high mitotic count (8/10 HPFs) and marked nuclear pleomorphism did not allow a formal exclusion of a mixed tumor with both PSTT and choriocarcinoma components.

**FIGURE 2:**
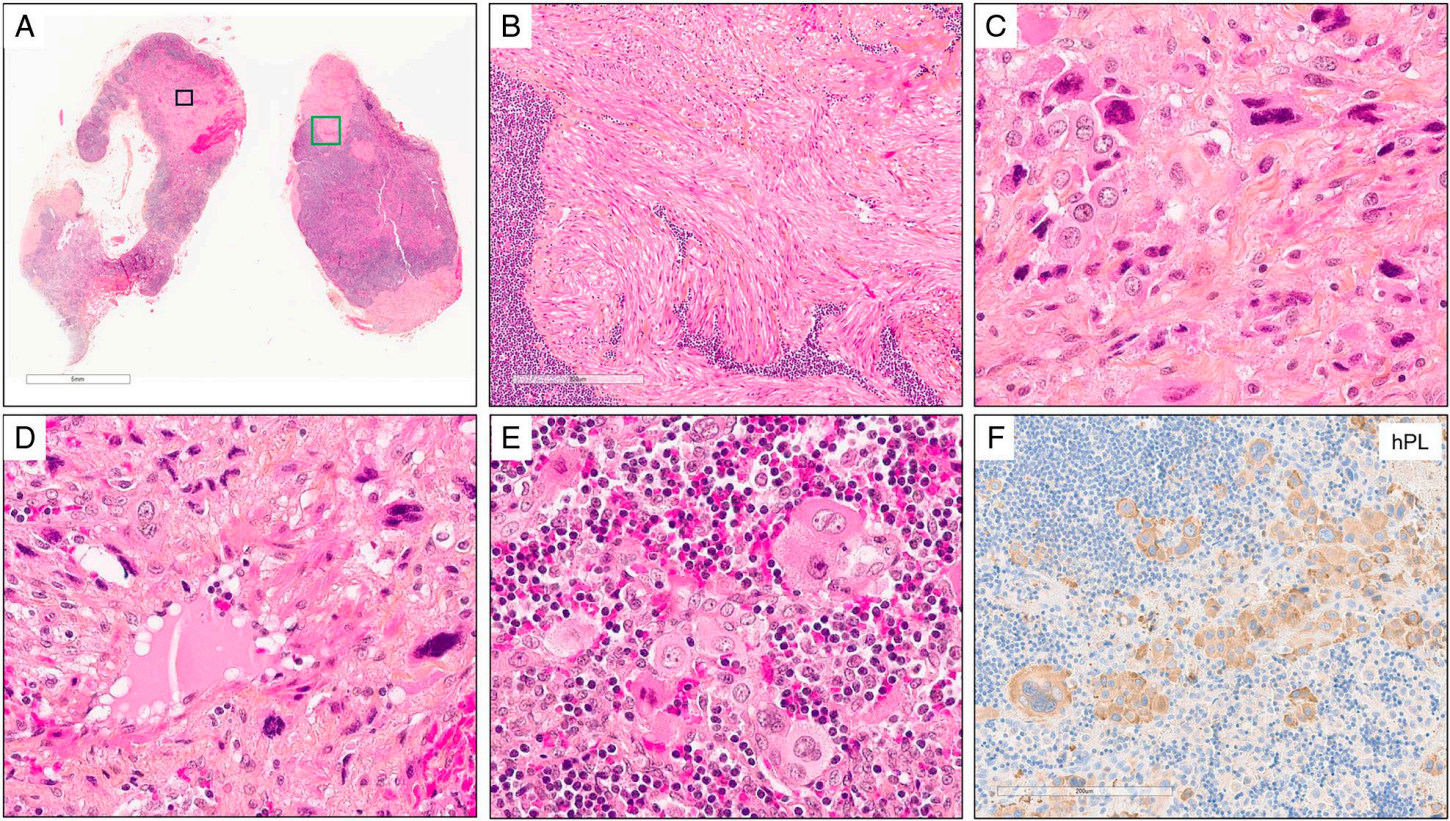
Histopathologic and immunohistochemical features of metastatic nongestational PSTTs. A, Pelvic lymph node: the green frame corresponds to picture (B) and the black frame to picture (C) (hematoxylin-eosin-saffron [HES], tumor #1). B Incidental nodal lymphangioleiomyomatosis (tumor cells expressed desmin, h-caldesmon, HMB45 [data not shown]) (HES, tumor #1). C, Tumor cells with abundant eosinophilic cytoplasm and pleomorphic atypical nuclei within fibrous tumor stromal reaction (HES, tumor #1). D, Tumor cells infiltrating a vascular wall (HES, tumor #1). E, Scattered tumor cells in a lymphocytic background (lymph node parenchyma), without fibrous tumor stromal reaction (HES, tumor #1). F, Diffuse positivity of hPL in tumor cells (hPL IHC, tumor #1). IHC indicates immunohistochemistry; PSTTs, placental site trophoblastic tumors.

In lymph node and other metastatic sites, morphology and immunostaining were similar to that of the uterine neoplasm, with sheets or nests of malignant ISIT and vascular invasion. Either scattered infiltrative tumor cells admixed within the metastatic site parenchyma, or some focal tumor cells embedded within fibrous tumor stromal reaction, were observed (Fig. 2). Of note, incidental nodal lymphangioleiomyomatosis was observed for patient #1.

### Molecular Findings

**FIGURE 3:**
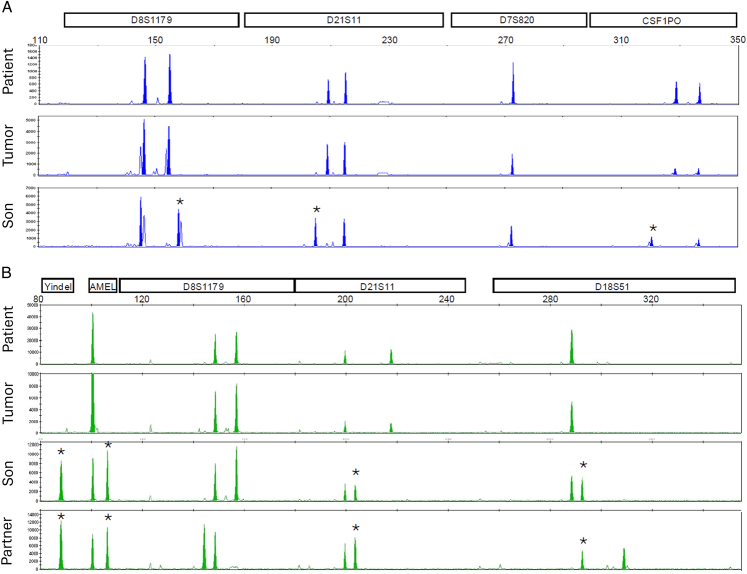
Representative images of STR genotyping of cases #2 and #4. In all cases, the alleles present in the tumor match those of the patient. In cases #2 (A) and #4 (B), none of the paternal alleles (*) in the patient’s child were present in the tumor. The *x*-axis represents fragment size. The *y*-axis represents arbitrary units of fluorescence. STR indicates short tandem repeat.

STR genotyping of all cases identified that the alleles in the tumor matched those of the patient, indicating a nongestational origin. Supporting the nongestational origin, the genotype of the child from patient #4’s only known pregnancy (Fig. 3) and the child from patient #2’s antecedent pregnancy did not match the respective tumors.

Multiple measures were taken to exclude the possibility that the absence of paternal alleles was due to high levels of contamination with patient cells. Multi-region sampling was performed in 4/5 (80%) tumors, with consistent results. A sensitive NGS-based SNP genotyping method was additionally used for 2 tumors (#3 and #4), which confirmed the absence of nonpatient/paternal alleles in the tumors (data not shown). Allelic imbalances at heterozygous loci infer the presence of copy number alterations in tumors and act as an internal control for the purity of the tumor sample. Allelic imbalances were detected at 1 to 3 STR loci in 3/5 (60%) tumors (Table 3). None of the loci with the identified imbalances, including the 12p locus *D12S391* detected in tumor #5, had imbalances in more than one tumor.

**TABLE 3:** Molecular Results of Nongestational Placental Site Trophoblastic Tumors

Patient/tumor #	Patient/tumor #1	Patient/tumor #2	Patient/tumor #3	Patient/tumor #4	Patient/tumor #5
No. distinct tumor areas studied by STR genotyping	2	2	4	4	1
Results of STR genotyping	No nonpatient alleles in the tumor	No nonpatient alleles in the tumor	No nonpatient alleles in the tumor	No nonpatient alleles in the tumor	No nonpatient alleles in the tumor
Results of SNP genotyping	NP	NP	No nonpatient alleles in the tumor	No nonpatient alleles in the tumor	NP
Paternal or child sample tested	NP	Child (nonmatch)	NP	Partner and child (nonmatch)	NP
Zygosity	Heterozygous	Heterozygous	Heterozygous	Heterozygous	Heterozygous
Allelic imbalance	*TH01* (chr 11), *D1S1656* (chr 1), *D16S539* (chr 16)	No consistent imbalances	*D5S818* (chr 5)	No consistent imbalances	*D12S391* (chr 12), *D22S1045* (chr 22)
aCGH	Loss of chr 8, 11, 16, and 22; Gain of chr 1q and 20	NP	NP	NP	NP
NGS (DNA)	*ERCC2* c.1752C>G; VAF: 42.6%	NP	NP	NP	NP
RNA-sequencing	No fusion-transcript	NP	NP	NP	NP

# indicates number; aCGH, array-comparative genomic hybridization; Chr, chromosome; NGS, next-generation sequencing; NP, not performed; SNP, single nucleotide polymorphisms; STR, short tandem repeat; VAF, variant allele frequency.

**FIGURE 4:**
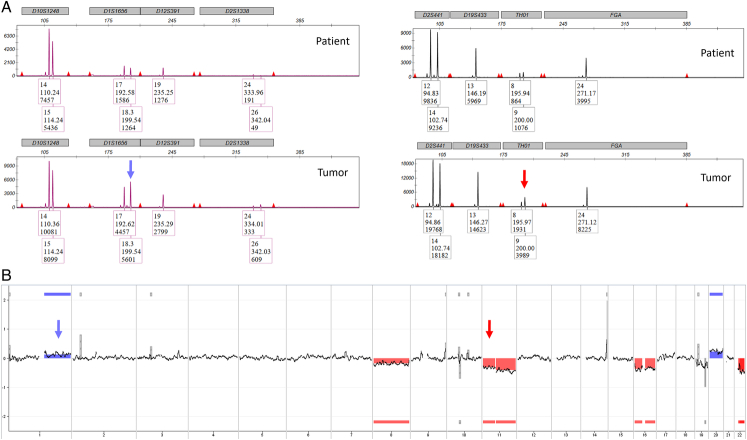
Examples of allelic imbalance and array-CGH analysis (case #1). A, STR genotyping identified that the alleles in the tumor were identical to those of the patient, indicating a nongestational origin. In addition, allelic imbalances at heterozygous loci infer the presence of copy number alterations in tumors (eg: *TH01* [red arrow] and *D1S1656* [blue arrow]). The *x*-axis represents fragment size. The *y*-axis represents arbitrary units of fluorescence. B, Array-CGH confirmed that the allelic imbalances identified were due to copy number alterations: the imbalances at *TH01* and *D16S539* due to loss of chromosome 11 (red arrow) and 16, respectively, and the imbalance at *D1S1656* due to gain of chromosome 1q (blue arrow). Additional chromosomal copy number alterations, at loci not covered or homozygous in the STR genotyping, were identified by array-CGH analysis: loss of chromosomes 8 and 22 and gain of chromosome 20. CGH indicates comparative genomic hybridization; STR, short tandem repeat.

Additional molecular profiling of tumor #1 was performed (Table 3). Array CGH confirmed that the allelic imbalances identified in the STR genotyping were due to copy number alterations: the imbalances at *TH01* and *D16S539* due to loss of chromosomes 11 and 16, respectively, and that the imbalance at *D1S1656* due to gain of chromosome 1q (Fig. 4). Additional chromosomal copy number alterations, at loci not covered or homozygous in the STR genotyping, were identified by array CGH: loss of chromosome 22 and gain of chromosome 20. Targeted-NGS of 100 solid tumor-associated genes identified a predicted loss-of-function variant in *ERCC2* (c.1752C>G [p.Y584*]; variant allele frequency of 42.6%) and no evidence for microsatellite instability. No transcript fusion was identified using RNA-sequencing.

### Treatment and Follow-Up

All patients were treated as per local guidelines for GTN. Two of the 5 (40%) patients received neoadjuvant chemotherapy, and 4/5 (80%) patients were treated by hysterectomy (patient #3 died almost immediately following PSTT diagnosis), with additional salpingo-oophorectomy(ies) for 2/5 (40%) patients, and lymph node dissection for one patient (1/5, 20%). After the surgical procedure, all patients (4/4, 100%) received adjuvant chemotherapy or immunotherapy because of positive lymph node(s), or uterine serosal involvement, or an interval >48 months between the end of the pregnancy and the diagnosis of PSTT (since tumors were initially supposed to be of gestational origin). The median PFS was 18 months (range: 0 to 85). Two of the 5 (40%) patients died from the disease.

## DISCUSSION

The present study has reported for the first time a series of 5 uterine PSTTs that were nongestational in origin. These tumors were initially treated as gestational tumors in origin due to their occurrence predominantly in women of child-bearing age (median of 32 y old) and due to their uterine location, their typical morphology and immunohistochemical profile, and a history of pregnancy for all patients. In all cases, the morphologic and immunohistochemical features, in both primary and metastatic sites, appeared consistent with those of gestational PSTTs.^1^ In the same way as gestational and nongestational choriocarcinomas are recognized as different entities in the *WHO Classification of Female Genital Tumor* 5th edition,^1^ we suggest that nongestational PSTTs could represent a distinct entity from their gestational counterpart, although more data and cases are required to confirm this hypothesis. As this tumor cannot be identified by pathologic features alone, it implies that STR genotyping should be performed systematically in all cases of PSTTs to exclude a nongestational origin.

Until recently, tumors demonstrating typical morphology and immunophenotype for PSTTs were assumed to be of gestational origin. Those found in extrauterine sites, such as the adnexa or pelvic locations, were proposed to be metastases or derived from an ectopic pregnancy.^16^ However, few of these extrauterine PSTTs were genotyped,^17^ and some nongenotyped extrauterine PSTTs could in fact be nongestational. Xing et al^18^ have reported 3 nongestational PSTTs among a series of 6 ovarian intermediate trophoblastic tumors. Subsequently, Shahi et al^19^ reported a further case of ovarian nongestational PSTT with lung metastasis. Very recently, McChesney et al^20^ have reported another ovarian nongestational PSTT in a 13-year-old female. These 5 female nongestational PSTTs were closely associated with ovarian mature teratoma,^18–21^ one of them with an identical homozygous genome in both the tumor and teratoma, which would support a germ cell origin.^22^ The nongestational origin was proven by molecular genotyping in 4 tumors, whereas one occurred in a 30-month-old girl, excluding a gestational origin.^21^ In the testis, nongestational PSTTs and ETTs are recognized as distinct entities.^23^ To date, 7 PSTTs have been reported in the testis or in metastatic sites in male, never as pure tumors, and often representing a low proportion of the main tumor volume, mainly composed of teratoma or other germ cell tumor type; this explains their classification within the “nonseminomatous germ cell tumors” category.^23–30^

The cellular origin of the PSTTs in this study is currently unclear. The absence of a germ cell tumor component in these tumors does not support a germ cell origin. Partial or complete homozygosity is a common feature of ovarian germ cell tumors^31^ but all 5 nongestational PSTTs were heterozygous herein. Chromosome 12 alterations (ie, isochromosome 12p, 12p gain) are present in most germ cell tumors^32^ and allelic imbalance at chromosome 12p was detected by STR genotyping in one of the five tumors (20%), but this method is not diagnostic for 12p gains and does not distinguish between gains and losses.

**FIGURE 5:**
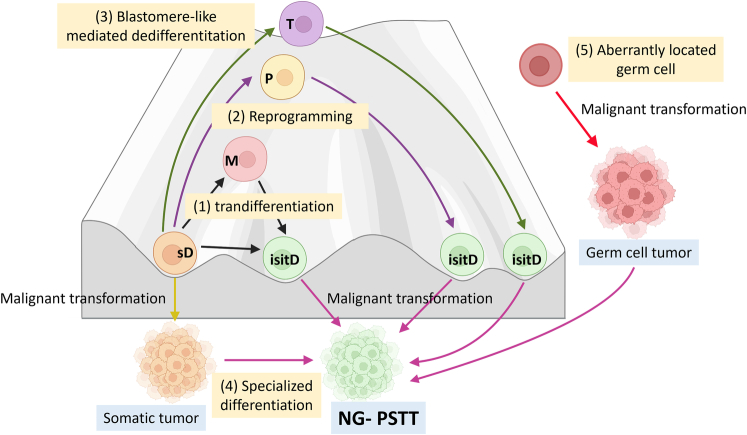
Schematic diagram illustrating hypotheses to explain the genesis of nongestational PSTTs. In the center, Waddington’s landscape (in gray) shows the plasticity of somatically differentiated cells. Several factors, such as environmental stresses (chronic inflammation, infectious diseases, chemical, or radiation exposure), hormonal variation, together with intrinsic epigenetic or genetic alterations, may trigger a dedifferentiation program of mature/terminally differentiated somatic cells and nongestational PSTT genesis *via* several pathways. Transdifferentiation (1) refers to the conversion of one terminally differentiated cell (eg, epithelial cell, smooth muscle cell) into another (eg, implantation site intermediate trophoblast [ISIT]). This process may be indirect with an intermediate precursor stage or direct. Reprogramming (2) refers to dedifferentiation of a terminally differentiated cell into a pluripotent cell that can differentiate into any other cell type (eg, ISIT). Blastomere-like mediated dedifferentiation pathway (3) refers to dedifferentiation to morula/blastocyst at a totipotent stage, and then direct differentiation into ISIT from trophectoderm. These events are then followed by the malignant transformation of ISIT into nongestational PSTT. At the bottom, specialized differentiation (4) refers to differentiation of malignant somatic cells (cancer stem cells mostly from undifferentiated or high-grade carcinoma) into malignant trophoblastic cells (nongestational PSTT). Finally, on the right side, an aberrantly located germ cell (5) gives rise to a germ cell tumor with a trophoblastic component. D indicates differentiated cell; isitD, implantation site intermediate trophoblast differentiation; M, multipotent cell (adult stem cell); NG-PSTT, nongestational placental site trophoblastic tumor; P, pluripotent cell (inner cell mass/embryonic stem cell, embryonic germ cell); sD, mature somatic cell differentiation; T, totipotent cell (zygote).

Alternatively, transdifferentiation or dedifferentiation/reprogramming processes of somatic cells, possibly following epigenetic modifications, may explain the genesis of the tumors reported in the present study.^33–36^ Liu has postulated that a “blastomere-like program” could lead to the transformation of some damaged/aged mature somatic cells,^37,38^ for which successive steps mimicking the preimplantation embryo occur along with erasing of the epigenetic marks from the parentally derived genome. Direct and specialized differentiation of malignant somatic cells into trophoblastic cells could also be another hypothesis. Although choriocarcinomatous differentiation is the most frequent,^6^ differentiation into ISIT or CTIT should also be considered. However, in the uterus, such differentiation usually occurs from a high-grade endometrial carcinoma component,^1,6,39^ which was excluded in the present study by a broad sampling of the tumors and an expert review of the slides. These different mechanisms of genesis are illustrated in Figure 5. Molecular profiling of additional tumors will be required to identify the cell of origin and highlight the molecular mechanisms of malignant transformation.

The identification of primary uterine nongestational PSTTs has several important clinical consequences. Firstly, the diagnosis of all PSTTs should include genotyping to determine the gestational or nongestational origin. Genotyping of PSTTs and ETTs is not routinely performed, except in cases where specific identification of the causative pregnancy is required.^40^ Thus, nongestational PSTTs are likely to be underdiagnosed. Identification of additional cases is required to delineate the features and clinical outcomes associated with this entity. Secondly, in surgical pathology practice, some morphologic differential diagnoses should be ruled out, including undifferentiated/dedifferentiated endometrial carcinoma, epithelioid leiomyosarcoma, endometrial stromal sarcoma, PEComa, and even melanoma.^1^ This requires a broad sampling of the surgical specimen and a double review of the slides by expert gynecologic and trophoblastic disease pathologists. Thirdly, the diagnosis could impact the prognosis of patients. Nongestational choriocarcinomas have a less favorable prognosis than gestational choriocarcinomas.^6^ Similarly, the clinical behavior of nongestational PSTTs reported herein appeared quite aggressive, with metastatic disease from the start (distant and/or lymph node metastases) and/or locally advanced disease in 80% of the patients, although 60% of them would have been classified as FIGO stage I using the FIGO staging for GTN.^12^ In total, 60% (6/10) of the female patients with nongestational PSTTs published^18–21^ and reported herein had metastatic disease at presentation or during follow-up, which is higher than in a large cohort of assumed or confirmed gestational PSTTs (37%).^8^ As these tumors were finally classified as nongestational, the retrospective use of FIGO staging for nongestational uterine tumors^1^ appears more appropriate to specify the risk of progression, especially because it includes the staging according to lymph node involvement. Moreover, 40% of the patients (#3 and #5) reported herein finally died from the disease. These patients had metastases at presentation and a median follow-up of 9 months. Even if PSTTs were nongestational herein, the pregnancy interval was ≥48 months in both cases, which means that these tumors would have belonged to the “poor prognosis” category at presentation, blinded to the STR genotyping result.^8^ In this category, the expected overall survival at 1-year follow-up is around 75% for gestational PSTT^8^ and was also 75% in the present study. However, more data are required to fully characterize the clinical behavior of nongestational PSTTs. Identification of additional cases and documentation in the International Database for Rare Trophoblastic Tumours (https://stdc.sites.sheffield.ac.uk/clinicians/idrtt-database) would help to establish the clinical prognosis for nongestational PSTTs.

Surgical resection of lesions is probably the treatment of choice in both gestational and nongestational PSTTs, adjuvant chemotherapy being usually restricted to patients with widespread tumors and/or long-interval between the antecedent/causative pregnancy and tumor diagnosis.^2^ In our study, all patients were initially managed as patients with gestational PSTTs, including hysterectomy in all patients but one, who died soon after PSTT diagnosis. Moreover, most of the patients who underwent chemotherapy, including a platinum-based regimen, were cured. Furthermore, similar to the majority of GTN,^6,41^ diffuse PD-L1 immunostaining was found in tumor #1, but also in one ovarian nongestational PSTT reported,^20^ suggesting immunotherapy as a therapeutic option. Interestingly, patient #2 progressed initially despite multi-agent chemotherapy but was in complete remission after hysterectomy and anti-PD-L1 therapy (*pembrolizumab*). Finally, although GTN usually shows an absence of high mutational burden and a microsatellite stable status,^42^ molecular testing should be considered in nongestational PSTTs to identify molecular targets, especially in advanced stages. For instance, pathogenic variants in *ERCC2*, such as those identified in tumor #1 herein, have been demonstrated to drive cisplatin sensitivity in muscle-invasive bladder cancers.^43^ Interestingly, the patient is free of disease 3 years after the diagnosis, following platinum-based chemotherapy.

The present study has limitations. Firstly, the small number of patients included and limited follow-up prevent a reliable comparison between nongestational PSTT and its gestational counterpart. However, uterine nongestational PSTTs are exceptionally rare tumors, and this series is the result of a European collaboration,^11^ a major strength of the present study. Secondly, we were unable to perform additional testing of tumor #5 to confirm the alteration found by STR genotyping using another molecular technique. Although chromosome 12 alterations (ie, isochromosome 12p, 12p gain) are commonly encountered in most germ cell tumors,^32^ they are not specific of germ-cell origin and have been reported in other histopathologic subtypes, including carcinomas and sex-cord stromal tumors.^44–46^ Interestingly, isochromosome 12p has been also found in uterine/ovarian carcinoma components of tumors with mixed epithelial and germ cell features (including choriocarcinomas), which are assumed to be somatically derived.^47,48^ Thus, even if 12p alteration had been identified in the present tumors, this would not have allowed us to conclude a germ cell origin.

To conclude, this study describes for the first time 5 nongestational PSTTs occurring as primary uterine tumors, which could represent a distinct entity. Diagnosis is reliant on molecular genotyping, as the tumor cells demonstrated the same morphologic, immunohistochemical, and pathophysiologic properties (infiltrative spread, vascular invasion, hCG secretion) as gestational PSTTs.

## Supplementary Material
