# Supplementary material for: Primary Uterine Nongestational Placental Site Trophoblastic Tumor as a Distinct Entity: A Report of 5 Cases
Source: Am J Surg Pathol. 2026 Jan 6;50(4):435–47. doi: 10.1097/PAS.0000000000002502 (PMC12978710; doi:10.1097/PAS.0000000000002502)
Supplement: Supplementary file 1 [file pas-50-435-s001.docx]

**Supplementary Digital Table S1**. Targeted DNA-Next generation sequencing panel.

| **Genes** | **Transcript** | **Exons/regions sequenced** |
| --- | --- | --- |
| *AKT1* | NM_001014431, NM_005163 | 2-12 |
| *ALK* | NM_004304 | 20-28 |
| *AR* | NM_000044 | full CDS |
| *ARAF* | NM_001654, NM_001256196 | 7,10-16 |
| *ARID1A* | NM_006015 | full CDS |
| *ARID2* | NM_152641 | full CDS |
| *ATM* | NM_000051 | 2-63 |
| *ATRX* | NM_000489 | full CDS |
| *B2M* | NM_004048 | full CDS |
| *BAP1* | NM_004656 | full CDS |
| *BARD1* | NM_000465 | full CDS |
| *BRAF* | NM_004333 | 6,11-18 |
| *BRCA1* | NM_007294 | full CDS |
| *BRCA2* | NM_000059 | 2-27 |
| *BRIP1* | NM_032043 | 2-20 |
| *CARD11* | NM_001324281 | 4-16 |
| *CCND1* | NM_053056 | full CDS |
| *CCNE1* | NM_001238 | 6-11 |
| *CDK4* | NM_000075 | full CDS |
| *CDK6* | NM_001145306, NM_001259 | full CDS |
| *CDK12* | NM_015083 | full CDS |
| *CDKN2A* | NM_000077 | full CDS |
| *CDKN2B* | NM_004936, NM_078487 | full CDS |
| *CHEK1* | NM_001114121 | 2-13 |
| *CHEK2* | NM_001005735 | 2,4-16 |
| *CTNNB1* | NM_001904 | 3,7-8,10-15 |
| *DAXX* | NM_004448 | full CDS |
| *DDR2* | NM_001014796 | 14-19 |
| *EGFR* | NM_005228 | 2-4,6-9,15-26 |
| *EIF1AX* | NM_001412 | 1-2 |
| *ERBB2* | NM_004448 | 6-8,12,16-24 |
| *ERBB3* | NM_001982 | 2-12,18-24 |
| *ERBB4* | NM_005235, NM_001042599 | 5-8,13-16,18-24 |
| *ERCC2* | NM_000400 | full CDS |
| *ESR1* | NM_000125 | 4-8 |
| *EZH2* | NM_004456 | full CDS |
| *FANCA* | NM_000135, NM_001286167 | full CDS |
| *FANCL* | NM_0018062 | full CDS |
| *FBXW7* | NM_0010101341 | full CDS |
| *FGFR1* | NM_023110 | 11-17 |
| *FGFR2* | NM_000141 | 6-11b,13-19 |
| *FGFR3* | NM_000142 | 7-10,12-18 |
| *GATA3* | NM_001002295 | full CDS |
| *GNA11* | NM_002067 | full CDS |
| *GNAQ* | NM_002072 | full CDS |
| *GNAS* | NM_000516 | full CDS |
| *HRAS* | NM_001130442, NM_005343 | 2-5 |
| *IDH1* | NM_001282386, NM_005896, NM_001282387 | 4 |
| *IDH2* | NM_002168 | 4 |
| *JAK1* | NM_001320923 | full CDS |
| *JAK2* | NM_001322194 | full CDS |
| *KDM6A* | NM_001291415 | full CDS |
| *KEAP1* | NM_012289, NM_203500 | full CDS |
| *KIT* | NM_000222 | 8-20 |
| *KMT2C* | NM_170606 | full CDS |
| *KRAS* | NM_033360, NM_033360 | 2-5 |
| *MAP2K1* | NM_002755 | 2-11 |
| *MAP2K2* | NM_030662 | 2-11 |
| *MC1R* | NM_002386 | full CDS |
| *MDM2* | NM_00145337 | full CDS |
| *MET* | NM_001127500 | 2,13-21 (+ intron 13-14) |
| *MITF* | NM_000248 | full CDS |
| *MTOR* | NM_004958 | 29-56 |
| *MYC* | NM_002467 | full CDS |
| *MYCN* | NM_ 001293228 | full CDS |
| *NBN* | NM_002485, NM_001024688 | full CDS |
| *NCK1* | NM_001291999, NM_006153 | full CDS |
| *NF1* | NM_001042492, NM_000267 | full CDS |
| *NOTCH1* | NM_017617 | full CDS |
| *NOTCH2* | NM_024408 | full CDS |
| *NRAS* | NM_002524 | 2-5 |
| *NTRK1* | NM_002529 | 15-19 |
| *NTRK3* | NM_001012338 | 15-20 |
| *PALB2* | NM_024675 | 1-13 |
| *PBRM1* | NM_018313 | full CDS |
| *PDGFRA* | NM_006206, NM_001347829 | 5-7,10-21 |
| *PIK3CA* | NM_006218 | 2,5-21 |
| *PIK3R1* | NM_181523 | full CDS |
| *POLD1* | NM_001256849 | 4-12 |
| *POLE* | NM_006231 | 3-14 |
| *PPP2R2A* | NM_001177591 | 1-10 |
| *PTCH1* | NM_000264 | full CDS |
| *PTEN* | NM_000314 | full CDS |
| *PTPN11* | NM_002834 | 2-3,7-13 |
| *RAC1* | NM_006908, NM_018890 | full CDS |
| *RAD51B* | NM_133510 | 2-11 |
| *RAD51C* | NM_0058276 | 1-9 |
| *RAD51D* | NM_002878 | 1-10 |
| *RAD54L* | NM_001142548 | 2-19 |
| *RAF1* | NM_002880 | 7,10-17 |
| *RB1* | NM_000321 | full CDS |
| *RET* | NM_020975 | 8-18 |
| *ROS1* | NM_002944 | 36-42 |
| *SF3B1* | NM_012433 | 13-18 |
| *SMAD4* | NM_005359 | full CDS |
| *SMARCA4* | NM_001128844 | full CDS |
| *STK11* | NM_000455 | full CDS |
| *TSC1* | NM_000368, NM_001162426 | full CDS |
| *TSC2* | NM_000548, NM_001318832 | full CDS |
| *VHL* | NM_000551 | full CDS |

CDS : coding DNA sequence
