# Supplementary material for: Primary Uterine Nongestational Placental Site Trophoblastic Tumor as a Distinct Entity: A Report of 5 Cases
Source: Am J Surg Pathol. 2026 Jan 6;50(4):435–47. doi: 10.1097/PAS.0000000000002502 (PMC12978710; doi:10.1097/PAS.0000000000002502)
Supplement: Supplementary file 2 [file pas-50-435-s002.docx]

**Supplementary Digital Table S2**. Targeted RNA-sequencing fusion transcript panel.

| **Genes** | **Transcripts** |
| --- | --- |
| *ACVR2A* | NM_001616, NM_001278579 ex1-4 |
| *AKT1* | NM_005163, NM_001014432 ex2-8,10-11 |
| *AKT2* | NM_001626, NM_001330511 ex1-2,4-5 |
| *AKT3* | NM_005465, NM_001206729 ex1-5,7-10 |
| *ALK* | NM_004304 |
| *ALPK1* | NM_025144, NM_001253884 ex3-4,10-13 |
| *AR* | NM_000044, NM_001011645 ex1-3,5-8 |
| *ARHGAP26* | NM_015071 ex2,10-12 |
| *ARHGAP6* | NM_006125 ex2 |
| *AXL* | NM_021913, NM_001278599 ex7-8,10-11,15-20 |
| *BCOR* | NM_001123385, NM_017745 ex 2-8,11-12,14-15 |
| *BRAF* | NM_004333 |
| *BRD3* | NM_007371 ex11-12 |
| *BRD4* | NM_058243 ex11-12 |
| *CAMTA1* | NM_015215 ex3,8-10 |
| *CCNB3* | NM_033031 ex2-4,6-7 |
| *CCND1* | NM_053056 |
| *CD274* | NM_014143, NM_001267706 ex 2,5 |
| *CDKN2A* | NM_000077 ex1 |
| *CHMP2A* | NM_014453 ex2-5 |
| *CIC* | NM_015125, NM_001304815 ex 12-13,17-21 |
| *CRTC1* | NM_015321, NM_001098482 ex1-6 |
| *CSF1* | NM_000757, NM_172210 ex1-5,7-8 |
| *CSF1R* | NM_005211 ex11-13 |
| *CTNNB1* | NM_001904, NM_001330729 ex2-5 |
| *DHX8* | NM_001322219 ex23 |
| *DICER1* | NM_030621, NM_177438, NM_001195573 ex23-28 |
| *DNAJB1* | NM_006145 |
| *EGF* | NM_001963, NM_001178131 ex15-19 |
| *EGFR* | NM_005228, NM_001346941 ex1-27 |
| *EPC1* | NM_025209 ex9,11 |
| *ERBB2* | NM_004448, NM_001289936 ex1-2,4-30 |
| *ERBB4* | NM_005235 ex2,4,14-18,23 |
| *ERG* | NM_004449, NM_001136154 ex3,5,7,9-11 |
| *ESR1* | NM_000125, NM_001122740, NM_001122741, NM_001122742, NM_001291230, NM_001291241, NM_001328100 |
| *ESRRA* | NM_004451 ex3 |
| *ETV1* | NM_004956, NM_001163147 ex2-7,9-13 |
| *ETV4* | NM_001986 ex1-10, NM_001261437, NM_001261439 |
| *ETV5* | NM_004454 ex2-3,9 |
| *ETV6* | NM_001987 ex1-4,6.7 |
| *EWSR1* | NM_005243, NM_013986, NM_001163285, NM_001163286, NM_001163287 |
| *FGF1* | NM_000800, NM_033136, NM_033137, NM_001144892, NM_001144934, NM_001144935, NM_001257205, NM_001257206, NM_001257207, NM_001257208, NM_001257209, NM_001257210, NM_001257211, NM_001257212 |
| *FGFR1* | NM_015850, NM_023105, NM_023106, NM_023110, NM_001174063, NM_001174064, NM_001174065, NM_001174066, NM_001174067 |
| *FGFR2* | NM_000141, NM_022970, NM_023029, NM_001144913, NM_001144914, NM_001144915, NM_001144916, NM_001144917, NM_001144918, NM_001144919, NM_001320654, NM_001320658 |
| *FGFR3* | NM_000142, NM_022965, NM_001163213 |
| *FGR* | NM_005248 |
| *FOS* | NM_005252 |
| *FOSB* | NM_006732, NM_001114171 |
| *FOXO1* | NM_002015 |
| *FOXO4* | NM_005938, NM_001170931 |
| *FOXR2* | NM_198451 |
| *FUS* | NM_004960, NM_001170634, NM_001170937 |
| *GLI1* | NM_005269, NM_001160045, NM_001167609 |
| *GRB7* | NM_005310, NM_001030002, NM_001242442, NM_001242443, NM_001330207 |
| *GREB1* | NM_014668, NM_033090, NM_148903 |
| *HMGA2* | NM_003483, NM_003484, NM_001300918, NM_001300919, NM_001330190 |
| *HRAS* | NM_005353, NM_176795, NM_001130442, NM_001318054 |
| *IDH1* | NM_005896, NM_001282386, NM_001282387 |
| *IDH2* | NM_002168, NM_001289910, NM_001290114 |
| *IGF1R* | NM_000875, NM_001291858 |
| *INSR* | NM_000208, NM_001079817 |
| *JAK2* | NM_004972, NM_001322194, NM_001322195, NM_001322196, NM_001322198, NM_001322199, NM_001322204 |
| *JAK3* | NM_000215 |
| *JAZF1* | NM_175061 |
| *KANSL1* | NM_015443, NM_001193465, NM_001193466 |
| *KIT* | NM_000222, NM_001093772 |
| *KRAS* | NM_004985, NM_033360 |
| *MAML2* | NM_032427 |
| *MAP2K1* | NM_002755 |
| *MAP3K8* | NM_005204, NM_001244134, NM_001320961 |
| *MAST1* | NM_014975 |
| *MAST2* | NM_015112, NM_001319245, NM_001324320, NM_001324321 |
| *MBTD1* | NM_017643 |
| *MDM2* | NM_002392, NM_001145337, NM_001145339, NM_001145340, NM_001278462 |
| *MEAF6* | NM_022756, NM_001270875, NM_001270876 |
| *MET* | NM_000245, NM_001127500, NM_001324401, NM_001324402 |
| *MGEA5* | NM_012215, NM_001142434 |
| *MKL2* | NM_014048, NM_001308142 |
| *MN1* | NM_002430 |
| *MSMB* | NM_002443, NM_138634 |
| *MUSK* | NM_005592, NM_001166280, NM_001166281 |
| *MYB* | NM_005375, NM_001130172, NM_001130173, NM_001161656, NM_001161657, NM_001161658, NM_001161659, NM_001161660 |
| *MYBL1* | NM_001080416, NM_001144755, NM_001294282 |
| *MYC* | NM_002467 |
| *MYOD1* | NM_002478 |
| *NCOA1* | NM_003743, NM_147223, NM_147233 |
| *NCOA2* | NM_006540, NM_001321703, NM_001321707, NM_001321711, NM_001321712, NM_001321713 |
| *NCOA3* | NM_006534, NM_181659, NM_001174087, NM_001174088 |
| *NFATC2* | NM_012340, NM_173091, NM_001136021, NM_001258292, NM_001258294, NM_001258295, NM_001258296, NM_001258297 |
| *NFE2L2* | NM_006164, NM_001145412, NM_001145413, NM_001313900, NM_001313901, NM_001313902, NM_001313903, NM_001313904 |
| *NFIB* | NM_001369458, NM_005596, NM_001190737, NM_001190738, NM_001282787 |
| *NOTCH1* | NM_017617 |
| *NOTCH2* | NM_024408, NM_001200001 |
| *NR4A3* | NM_006981, NM_173199, NM_173200 |
| *NRAS* | NM_002524 |
| *NRG1* | NM_013956, NM_013957, NM_013958, NM_013959, NM_013960, NM_013962, NM_013964, NM_001159995, NM_001159996, NM_001159999, NM_001160001, NM_001160002, NM_001160004, NM_001160005, NM_001160007, NM_001160008, NM_001322197, NM_001322201, NM_001322202, NM_001322205, NM_001322206, NM_001322207, NM_004495 |
| *NTRK1* | NM_001007792, NM_002529, NM_001012331 |
| *NTRK2* | NM_006180, NM_001007097, NM_001018064, NM_001018065, NM_001018066, NM_001291937 |
| *NTRK3* | NM_001320134, NM_001320135, NM_001012338, NM_001007156, NM_002530, NM_001243101 |
| *NUMBL* | NM_004756, NM_001289979, NM_001289980 |
| *NUTM1* | NM_175741, NM_001284292, NM_001284293 |
| *PAX3* | NM_000438, NM_013942, NM_181457, NM_181458, NM_181459, NM_181460, NM_181461, NM_001127366 |
| *PAX8* | NM_003466, NM_013952, NM_013953, NM_013992 |
| *PDGFB* | NM_002608, NM_033016 |
| *PDGFD* | NM_025208, NM_033135 |
| *PDGFRA* | NM_006206, NM_001347827, NM_001347828, NM_001347829, NM_001347830 |
| *PDGFRB* | NM_002609 |
| *PHF1* | NM_002636, NM_024165 |
| *PHKB* | NM_000293, NM_001031835 |
| *PIK3CA* | NM_006218 |
| *PKN1* | NM_002741, NM_213560 |
| *PLAG1* | NM_002655 |
| *PPARG* | NM_001330615, NM_005037, NM_138711, NM_138712, NM_015869 |
| *PRDM10* | NM_020228, NM_199437, NM_199438, NM_199439 |
| *PRKACA* | NM_002730, NM_207518, NM_001304349 |
| *PRKACB* | NM_182948, NM_002731, NM_207578, NM_001242857, NM_001242858, NM_001242859, NM_001242860, NM_001242861, NM_001242862, NM_001300915, NM_001300916, NM_001300917 |
| *PRKCA* | NM_002737 |
| *PRKCB* | NM_002738, NM_212535 |
| *PRKCD* | NM_006254, NM_212539, NM_001316327 |
| *PRKD1* | NM_002742, NM_001330069 |
| *PRKD2* | NM_016457, NM_001079880, NM_001079881, NM_001079882 |
| *PRKD3* | NM_005813 |
| *RAD51B* | NM_002877, NM_133509, NM_133510, NM_001321809, NM_001321810, NM_001321812, NM_001321814, NM_001321815, NM_001321817, NM_001321818, NM_001321819, NM_001321821 |
| *RAF1* | NM_002880 |
| *RELA* | NM_021975, NM_001145138, NM_001243984, NM_001243985 |
| *RET* | NM_020975, NM_020630 |
| *ROS1* | NM_002944 |
| *RSPO2* | NM_178565, NM_001282863, NM_001317942 |
| *RSPO3* | NM_032784 |
| *SS18* | NM_005637, NM_001308201, NM_001007559 |
| *SS18L1* | NM_198935, NM_001301778 |
| *STAT6* | NM_003153, NM_001178078, NM_001178079, NM_001178080, NM_001178081 |
| *TAF15* | NM_003487, NM_139215 |
| *TCF12* | NM_003205, NM_207036, NM_207037, NM_207038, NM_001322151, NM_001322152, NM_001322156, NM_001322157, NM_001322158, NM_001322159, NM_001322161, NM_001322162, NM_001322164, NM_001322165 |
| *TERT* | NM_198253, NM_001193376 |
| *TFE3* | NM_006521, NM_001282142 |
| *TFEB* | NM_007162, NM_001167827, NM_001271943, NM_001271944, NM_001271945 |
| *TFG* | NM_006070, NM_001007565, NM_001195478, NM_001195479 |
| *THADA* | NM_022065, NM_001083953, NM_001345923, NM_001345924, NM_001345925 |
| *TMPRSS2* | NM_001135099, NM_005656 |
| *USP6* | NM_004505, NM_001304284 |
| *VGLL2* | NM_153453, NM_182645 |
| *WWTR1* | NM_015472, NM_001168278, NM_001168280 |
| *YAP1* | NM_006106, NM_001130145, NM_001195044, NM_001195045, NM_001282097, NM_001282098, NM_001282099, NM_001282100, NM_001282101 |
| *YWHAE* | NM_006761 |
