# Supplementary material for: Primary Uterine Nongestational Placental Site Trophoblastic Tumor as a Distinct Entity: A Report of 5 Cases
Source: Am J Surg Pathol. 2026 Jan 6;50(4):435–47. doi: 10.1097/PAS.0000000000002502 (PMC12978710; doi:10.1097/PAS.0000000000002502)
Supplement: Supplementary file 3 [file pas-50-435-s003.docx]

**Supplementary Digital Table S3**. Clinical data, treatment, and follow-up.

| **Patient/tumor #** | **Patient/tumor #1** | **Patient/tumor #2** | **Patient/tumor #3** | **Patient/tumor #4** | **Patient/tumor #5** |
| --- | --- | --- | --- | --- | --- |
| **Sample available** (type, location) | Uterus and LN (right pelvic and inter-aortocaval LN) | Right salpingo-oophorectomy and partial removal of cornua; subsequently complete uterus | Endometrial curettage | Uterus, right and left salpingo-oophorectomies, omentum | Uterus (subtotal), left salpingo-oophorectomy |
| **Age at presentation** (years) | 34 | 28 | 25 | 45 | 32 |
| **Pregnancy/obstetrical history** (gravidity and parity, cesarean section) | G1P1, cesarean delivery | G2P1, normal delivery but retained products post-delivery requiring evacuation of retained products of conception | G1P0 | G1P1 | G3P3 |
| **Past medical/ surgical history** | Beckwith Wiedemann syndrome, bicornuate uterus | - | Hiatus Hernia, fibroadenoma | - | NA |
| **Clinical symptoms** | Amenorrhea for 10 months, recent metrorrhagia | Metrorrhagia positive pregnancy test | Fatigue, shortness of breath, chest pain, cough, vaginal discharge | Bloating, fatigue, and irregular vaginal bleeding for 8 months | Missed abortion (2^nd^ month) |
| **Time between end of pregnancy and diagnosis of PSTT** (months) | 10 | 72 | ~48 | 264 | 108 |
| **Serum hCG level at presentation** (mUI/mL) | 74 | 330 | 319 | 7 | 6571 mIU/mL |
| **Neoadjuvant treatment** | None | EP/EMA, PAC-E PAC-PLT MTX | Low dose EP, MTX | None | None |
| **Surgical treatment/ procedure** | Hysterectomy and sentinel node procedure, and then lumbo-aortic LN (22 LN; 1LN+/22 LN) | Hysterectomy + peritoneal and ovarian staging biopsies | NP | Hysterectomy, BSO, omentectomy | Subtotal hysterectomy and left salpingo-oophorectomy, followed by completed surgery with cervical extirpation |
| **Adjuvant treatment** | EP/EMA | Pembrolizumab | NP | EP/EMA | EP/EMA followed by TP-TE |
| **Primary location** | Uterine fundus and right uterine horn | Right cornua of the uterus | Uterus | Uterus | Uterus |
| **FIGO stage at diagnosis for GTD** | I | I | IV | II | I (and then IV) |
| **FIGO for non-gestational uterine tumor** | IIIC | IIIA | IVB | IIIB | IIIC (and then IVB) |
| **Uterine serosal involvement** | Yes | Yes | Yes | Yes | No |
| **Extra-uterine location(s)** | Right pelvic LN | None | Lung, liver, LN, breast, diaphragm, kidney, pericardium | Left parametrial involvement | Necrotic LN with stenosis of the distal third of the right ureter. Other pathological LN in the pelvis (more pronounced in the para-iliac and obturator space on the right), and bone secondary deposit within the symphysis of the pubis |
| **Follow-up duration** (months) | 36 | 85 | <1 | 18 | 18 |
| **Relapse / outcome after treatment completion** | None, NED | None, NED | DOD | None, NED | DOD |
| **PFS** (months) | 36 | 85 | 0 | 18 | NA |

#: Number; BSO: Bilateral salpingo-oophorectomy; DOD: Died of disease; EP/EMA: Etoposide and Cisplatin, and then Etoposide, Methotrexate, and Dactinomycin; FIGO: International federation of gynecology and obstetrics; GxPx: Gravidity x and parity x; hCG: Human chorionic gonadotrophin; LN: Lymph node; MTX: Methotrexate; NA: Not available; NED: No evidence of disease; NA: Not available; NP: Not performed; PAC-E: Cisplatin, Doxorubicin, Cyclophosphamide, Etoposide; PAC-PLT: Cisplatin, Doxorubicin, Cyclophosphamide, and platinum; PFS: progression free survival; PSTT: Placental site trophoblastic tumor; TP/TE: Paclitaxel/Cisplatin alternating with Paclitaxel/Etoposide.
