# Supplementary material for: Primary Uterine Nongestational Placental Site Trophoblastic Tumor as a Distinct Entity: A Report of 5 Cases
Source: Am J Surg Pathol. 2026 Jan 6;50(4):435–47. doi: 10.1097/PAS.0000000000002502 (PMC12978710; doi:10.1097/PAS.0000000000002502)
Supplement: Supplementary file 4 [file pas-50-435-s004.docx]

**Supplementary Digital Table S4**. Immunohistochemical results.

|  | **Patient/tumor #1** | **Patient/tumor #2** | **Patient/tumor #3** | **Patient/tumor #4** | **Patient/tumor #5** |
| --- | --- | --- | --- | --- | --- |
| **AE1/AE3** | + | NP | + | + | NP |
| **CAM 5.2** | NP | + | NP | + | NP |
| **CK18** | + | NP | NP | NP | + |
| **p63** | - | - (occasional cells) | - | - | - (occasional cells) |
| **p40** | - | NP | - | NP | NP |
| **hPL** | + | + (focal) | + | + | + (focal) |
| **hCG** | + (focal) | + (focal) | + (focal) | + (focal) | + (focal) |
| **Inhibin** | + | + | NP | + (focal) | NP |
| **Ki-67** | 18% | 10-15% | NP | 40% | 30% |
| **GATA3** | + | + | + | + | + |
| **Desmin** | - | NP | NP | - | NP |
| **Caldesmon** | - | NP | - | - | NP |
| **Smooth-muscle actin** | - | NP | - | - | NP |
| **MelanA** | - | NP | - | NP | NP |
| **HMB45** | - | NP | - | NP | NP |
| **SALL4** | - | NP | NP | NP | NP |
| **p53** | Wild-type | NP | NP | Wild-type | NP |
| **PAX8** | - | NP | NP | - | NP |
| **ER** | - | NP | NP | - | NP |
| **PR** | - | NP | NP | - | NP |
| **PD-L1** | 50% | NP | NP | NP | NP |
| **MMR proteins** | + (pMMR) | NP | NP | NP | NP |
| **PLAP** | + (focal) | + (focal) | NP | - | NP |
| **MCAM (CD146, Mel-CAM)** | NP | + | NP | NP | NP |
| **CD10** | + | + | NP | - | NP |

#: Number; +: Positive; -: Negative; NP: Not performed.
